# Defining the Role of the Fire and Rescue Service in Mental Health Support for Older Adults: A Qualitative Study

**DOI:** 10.1111/hex.70028

**Published:** 2024-09-19

**Authors:** Tamsin Fisher, Carolyn A. Chew‐Graham, Nadia Corp, Saeed Farooq, Paul Kingston, Ian Read, Gary Spolander, Jane Southam, Dean Stevens, Carmel Warren, Tom Kingstone

**Affiliations:** ^1^ School of Medicine Keele University Staffordshire UK; ^2^ Research and Innovation Department Midlands Partnership University NHS Foundation Trust Stafford UK; ^3^ Centre for Ageing and Mental Health, University of Chester Chester UK; ^4^ Staffordshire Fire and Rescue Service Staffordshire UK; ^5^ School of Applied Social Science Robert Gordon University Aberdeen UK

**Keywords:** anxiety and depression, Fire and Rescue Service, health and social care, mental health, non‐traditional providers, older adults

## Abstract

**Introduction:**

Anxiety and depression in older adults (60+ years of age) are under‐diagnosed and under‐treated. Older adults are less likely to seek help for these problems due to a lack of awareness, difficulty accessing health care due to availability or disability and fear of loss of independence. Existing points of contact between older adults and non‐traditional services, for example, the Fire and Rescue Service (FRS), could provide opportunities to support help‐seeking for mental ill‐health. The FRS conduct Home Fire Safety Visits (HFSVs) with older adults and are well positioned to provide health‐related support. This study examines a range of perspectives on the potential role of the FRS in the identification of, and signposting for, anxiety and depression in older adults.

**Methods:**

This was a qualitative study carried out using mixed methods in West Midlands, UK. Semi‐structured interviews were conducted with older adults and health and social care providers (practitioners, managers, commissioners) to explore the acceptability of the FRS expanding its role to detect and signpost for anxiety and depression in older adults. Observations examined delivery of existing HFSVs to older adults. Data were combined and analysed using a reflexive thematic approach.

**Results:**

Eighteen health and social care providers and 8 older adults were interviewed; 10 HFSVs were observed. Two overarching themes were identified: (1) Potential role for the FRS and (2) Operationalising identification of mental health problems by FRS. Interviews and observations demonstrated how HFSVs offer a suitable opportunity to start conversations about mental health. All interview participants felt that although the FRS would be well placed to deliver an intervention, they would require training, support and a referral pathway co‐produced with and supported by health and social care partners.

**Conclusion:**

A whole‐system approach is needed if the FRS are to expand HFSVs to identify mental health problems in older adults and provide signposting to appropriate services.

**Patient or Public Contribution:**

J.S. is a public co‐investigator. A Patient Advisory Group contributed to the initial funding application, design and conduct of the study, including data analysis and advice on dissemination.

## 1 Introduction

Mental health problems are a leading cause of disease burden in England [1]. Depression and anxiety are associated with disability [2], and can contribute to poorer health outcomes and increased mortality in people with long‐term physical conditions [3, 4]. Experiences of bereavement, loneliness, caring responsibilities and retirement all have a detrimental impact on mental health, contributing to depression, self‐harm and suicide [5, 6]. Physical long‐term conditions and psycho‐social factors place older adults (defined in this paper as 60 years of age and over) at risk of developing mental health problems, where appropriate systems of support are not available [7].

Anxiety and depression are as common in older adults as in younger adults [8], yet fewer older adults seek help to address mental health concerns [9]. Stigma, a fear of being a burden, and limited mental health awareness or literacy are just some of the barriers that older adults encounter [10, 11]. Further, symptoms of depression (i.e., low mood) may often be viewed by older adults as a ‘natural’ part of aging [12]. The NHS 5 Year Forward View for Mental Health aimed to improve access to mental health services in England; a specific target was set to expand access to services for 380,000 older adults with mental ill‐health and long‐term conditions by 2023/2024 [13]. Older adults, however, continue to be under‐represented in NHS Talking Therapy services (formerly known as Improving Access to Psychological Therapies, or IAPT) [14] and make up less than 6% of users of those services [15]. Barriers to help‐seeking seem to persist despite system‐wide attempts to improve access to services.

The community mental health framework for adults and older adults recognises the need to adopt place‐based models of care that utilise existing community assets to address inequalities in mental health [16]. Within this space, evidence for mental health interventions delivered by non‐traditional providers—public services that would not traditionally deliver health care interventions but who could act as key assets in this regard—demonstrates promise. For example, voluntary, community and social enterprises (VCSE) have shown it is feasible and acceptable to deliver psychosocial interventions for social isolation [17] and anxiety and depression [7]. A realist review described interventions provided by Fire and Rescue Services (FRSs) for a range of health problems and behaviours [18]. Current evidence suggests that FRSs can deliver interventions to identify loneliness, social isolation, alcohol dependency, smoking cessation, falls prevention [19, 20], and deliver covid vaccinations [21]. However, research in this area typically lacks methodological rigour [18].

**Figure 1:**
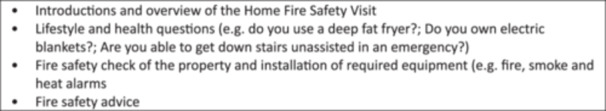
Outline of Home Fire Safety Visits (as observed).

The FRS seem well positioned to expand Its role as a non‐traditional provider; the service is trusted by the public [19], provides universal and non‐stigmatised services and FRS personnel have contact with people in their own homes. For example, more than 530,000 Home Fire Safety Visits (HFSVs) were delivered in 2022/2023 [22]. For context, HFSVs are delivered by Technicians and Community Safety Officers (CSOs) to people in their home and involve the provision of fire prevention advice, conducting safety checks, fitting equipment (e.g., fire alarms) (see Figure 1); visits can last up to 2 h depending on client needs.

The authors conducted a mixed methods study which aimed to explore the opportunity for, and acceptability of, adapting HFSVs to include detection and signposting for anxiety and depression in older adults. A paper is published elsewhere describing views from FRS staff [23]. The current paper reports views of other key stakeholders (older adults and health and social care [HSC] providers) on the potential expansion of the FRS role into mental health.

## 2 Methods

### 2.1 Research Design

Mixed methods [24] using semi‐structured interviews to explore the perspectives of a range of HSC providers about the acceptability of the FRS expanding their HFSVs to incorporate discussion about mental health, and observations to examine delivery of HFSVs in real‐time and identify opportunities for early detection of common mental health problems. Mixed methods were used to gather data from different perspectives and dimensions of experience to support triangulation between views of older adults and HSC providers and observations of HFSVs. This helped to develop an in‐depth understanding of different stakeholder needs, capabilities and perceptions of acceptability.

We used non‐participant observation [25] to better understand the role of the FRS HFSVs. Observations provided access to real‐time delivery of these home visits, to examine circumstances and settings in which visits took place, experience interactions between recipients and providers and to understand the flow of visits and opportunities for mental health promotion. Observations also generated data for comparison to be analysed against interview data. Observations were recorded in descriptive fieldnotes using a flexible framework (Supporting Infomation S1: Appendix 1).

Ethics approval was obtained from Keele University Research Ethics Committee [reference MH‐210200].

### 2.2 Research Setting

Recruitment took place in the West Midlands, UK. Data collection was completed between February 2022 and April 2023.

### 2.3 Recruitment

#### 2.3.1 Interviews

A purposive approach to sampling was applied to recruit a range of HSC providers for interviews from across specialist and community mental health, primary care, social care public health and VCSEs. Individuals were identified through professional networks; this approach was supplemented by snowballing techniques to identify prospective participants through existing ones [26]. Study information was distributed to prospective participants from the research team.

Older adults 60 years of age or over, with and without prior experience or knowledge of HFSVs, living at home in Staffordshire and who could speak English were identified through the FRS and community networks. An existing FRS client database was screened against a set of inclusion criteria (Age 60 years or older; Received HFSV in last 12 months; Living in their own home; Living in Staffordshire; English speaking). Information about the study was posted to clients by the FRS. People who were interested in participating contacted the research team by email or post. Older adults were also identified through community networks and social media (Facebook, Twitter). Interested individuals were directed to contact the research team by email or telephone; a member of the research team then sent further information and arranged an interview.

Consent was obtained from all participants before the start of the interview either using an online form, if conducted via video or telephone call, or a paper copy if the interview was conducted face‐to‐face.

#### 2.3.2 Observations

For observations of HFSVs, older adults who were due to receive a HFSV were identified by an FRS administrator. Summary information was provided to these prospective participants over the telephone by the FRS; an information sheet and consent form were then sent by post ahead of the HFSV. On booking the HFSV, three‐stage consent was followed, the FRS administrator gained verbal consent for T.F. to observe the visit; T.F. then confirmed verbal consent on the door step of the property on the day of the HFSV followed by written consent in the home from the recipient and provider at the beginning of the HFSV.

### 2.4 Data Generation

**Figure 2:**
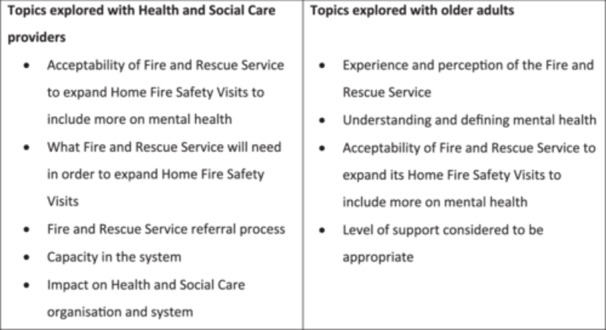
Content of topic guides for semi‐structured interviews.

Interviews were conducted by T.F. online via Microsoft Teams or in‐person at a location chosen by participant, such as their home. Interviews were semi‐structured and followed a topic guide developed from the literature with input from a Patient Advisory Group (PAG). The topic guides broadly covered the topics listed in Figure 2.

Observations of HFSVs were conducted by T.F. alongside the provider of the visit (FRS technician or CSO). Consent to participate was obtained before entry into the home from the householder and FRS staff member. Observations focused on: delivery of the HFSV (content and timeline), interaction between HFSV recipient and provider, communication, relationship development and level of engagement. Observations were recorded in a written fieldnote journal. We used a framework to outline key elements (see Supporting Information S1: Appendix 1 for framework).

### 2.5 Data Analysis

Interview data were transcribed verbatim and written fieldnotes were typed up. Data were analysed concurrently using a reflexive thematic approach [27] and each interview and observation informed subsequent data collection. A reflexive journal was used for observations which supported T.F. to acknowledge positionality, critically examine assumptions and document reflections that may inform the analytic process. All data was coded inductively by T.F. and organised into themes. HSC and older adult interview transcripts and observational data were analysed separately, at first, to support comparisons within each data set. All were then combined to support comparison across the data. T.F. led data analysis and held regular meetings with T.K. and C.A.C.‐G. to discuss and inform codes and themes. The PAG and all other authors reviewed a preliminary set of themes and corresponding data extracts to refine themes.

### 2.6 Patient and Public Involvement and Engagement

A PAG, comprising six public contributors with lived experiences of mental ill‐health (personal or as a caregiver), informed all aspects of the research. Contributors supported the design of participant documents, recruitment, topic guides, commented on analysis of data presented in this paper and supported the design and delivery of a dissemination workshop held in March 2023 with key HSC partners, researchers and FRS staff. Co‐author J.S. is a public co‐investigator.

## 3 Results

Interviews with 18 HSC providers and 12 older adults were completed. Interviews were conducted face‐to‐face (HSC interviews *n* = 2; older adult interviews *n* = 5) or online (HSC interviews *n* = 16; older adult interviews *n* = 7) depending on the participants' preference (e.g., in their home, place of work of at the study host institution). HSC occupations included social worker (*n* = 3, including one service manager), commissioner (*n* = 3), general practitioner (GP) (*n* = 2), community matron (*n* = 2), psychotherapist (charitable organisation) (*n* = 2 including one service manager), cognitive behavioural therapist (*n* = 1), senior lecturer in adult nursing (*n* = 1), community liaison manager (*n* = 1), mental health crisis team (*n* = 1), mental health nurse (*n* = 1) and social prescriber (*n* = 1). Participant characteristics are described in Tables 1, 2, 3. Data from four interviews (not included in the 12 above) with older adults were excluded before analysis due to the research team considering these people as ‘imposter’ participants [28]. Ten HSFV visits were observed; participant characters are described in Table 2.

**Table 1:** Health and Social Care provider interview participant characteristics.

Characteristic	Number of participants
Sex	
Male	7
Female	11
Age (years old)	
30–39	3
40–49	4
50–59	10
60–69	1
Ethnicity	
White British	17
Indian British	1
Length of time in role (years and months)	
No data	3
0–5 years	5
6–10 years	2
11–15 years	7
16–20 years	1
Qualification	
No degree	5
Advanced diploma	1
Bachelors	8
Masters	3
PhD	3

**Table 2:** Older adult interview participant characteristics.

Characteristic	Number of participants
Sex	
Male	1
Female	7
Age (years old)	
60–69	2
70–79	4
80–89	2
Ethnicity	
White British	7
Did not wish to disclose	1
Living status	
Alone	5
With others	3
Received a Home Fire Safety Visit	
Within the last 6 months	3
More than 6 months ago	1
Never	4

**Table 3:** Older adult Home Fire Safety Visit recipient characteristics.

Characteristic	Number of participants
Sex	
Male	7
Female	6
Age (years old)	
60–69	1
70–79	1
80–89	10
Living status	
Alone	7
With others	3
Reason for HFSV	
Routine check	1
Referral	5
Unknown	4

Two overarching themes are reported: (1) Potential role for the FRS with sub‐themes of ‘Eyes on the ground’ and ‘A time to talk’ and (2) Safely identifying mental health problems with sub‐themes of ‘Opening a can of worms’, ‘Preparing the public in advance’, ‘In need of training’ and ‘Making space for more referrals’.

Data extracts will be presented to support the analysis; each data extract is annotated to identify the participants pseudonym (e.g., OA01—Older Adult; HSC01—HSC professional), occupation or role in the study.

### 3.1 A Potential Role for the FRS

Most HSC providers endorsed the idea that FRS staff could support the detection of, and signposting for, anxiety and depression in older adults:

I just think that the more people do talk about it [mental health] and … the more they are raising their own awareness. There might be that odd patient who doesn't want to admit how they're feeling to a doctor or a nurse but might talk to the fire safety officer.

(HSC05, Community Matron)

It was not just acceptable for some HSC providers, but expected:

I don't actually think ‘acceptable’ is the term for me. I think there should be an expectation that there should be a push around mental health.

(HSC03, Senior Lecturer in Adult Nursing)

One older adult reported how the HFSV visit offered an opportunity for a discussion about mood, when they may not be able to talk to family and friends:

…maybe some people would just say to their family, ‘Yes I'm fine, I'm fine’, but really they're not you know? Just they don't want to be a nuisance, so don't want to bring it up. So maybe some people are better, I know at church some people are better talking to someone.

(OA10, 74 years old)

During one HSFV, it was observed how an opportunity to discuss mental health and inquire about help‐seeking arose during routine provision. The recipient (OA14) described feeling overwhelmed after their spouse had been hospitalised several times in recent months.

OA14 admitted that she was probably struggling more than she like to admit and that she almost lost OA13 twice when he was in hospital and that has taken its toll. CSO asked both older adults whether they had approached their GP at all about how they were both feeling. OA14 admitted that they'd raised it [feeling low] with GP for OA13, but not for herself and she probably should think about it. She commented that she just worries about OA13 to which CSO sympathised with.

(Observation data 18 October 2022)

The following sub‐themes address different dimensions and components of a potential role for FRS.

#### 3.1.1 Eyes on the Ground

Older adults generally spoke very highly of the FRS as a caring organisation that they trusted. Older adults acknowledged that the FRS staff were friendly and trustworthy. It was suggested that because they are not viewed traditionally as health and wellbeing experts, older people might feel more comfortable discussing their mental health without the stigma associated with it:

if you had a fire they'd come out and they'd make sure you're safe, and so it also goes along with your wellbeing. If they thought you were struggling at home or in an environment where you're not safe, or you don't feel safe, maybe they're a better person to speak to because you look at people like that, like the police, they take care of you. Fire and rescue they take care of you.

(OA10, 74 years old)

Some HSC providers reflected that some HSC services are being delivered remotely, in the wake of COVID‐19 restrictions, while the HFSV, conducted in the older people's home, offers the opportunity for a professional to see the person in their own surroundings. This was beneficial for the service user and their wellbeing:

It's all over the phone and a lot of the elderly population aren't happy to talk over the phone. They want to see somebody face to face… I think the fact that somebody's there with them, showing the time, it'll be good.

(HSC04, Community Matron)

HSC providers suggested the FRS are able to gain access to properties of service users, but also gain trust and therefore access to the lives of some potentially vulnerable service users:

The Fire Service can certainly reach people that perhaps we can't reach and as well, maybe other people services as well, like the Mental Health Team and that sort of thing.

(HSC02, Social Worker)

#### 3.1.2 A Time to Talk

HSCs described the impact of funding cuts on their own resources and a move towards telephone and online consultations instead of face‐to‐face appointments:

So you know, people will always get more satisfaction if they have a 20‐min appointment than a 10‐min appointment. And if you're then reducing it to a text that says, ‘Do this online’ you can imagine that actually people aren't going to engage because they don't feel they've been listened to.

(HSC08, GP)

Face‐to‐face contact may also help overcome other challenges (e.g., hearing loss):

…a lot of it is phone calls and a lot of elderly people they're deaf. They can't hear on the phone. That's why I think if they're [the fire service] going in they'll probably get a lot more information than a GP doing a phone consultation.

(HSC04, Community Matron)

One participant who worked in secondary care services felt that an intervention delivered by the FRS could support early detection and lead to the prevention of a mental health crisis:

It would help the service as well, because they wouldn't be coming in as crisis, maybe, in 2 months' time, where they haven't spoken up and then we're having to go out in an emergency…

(HSC14, Mental Health Nurse)

While the FRS staff may have more time with their service users than their partner agencies, some HSC providers and older adults felt that the FRS should ensure they approach the topic of mental health sensitively:

It would have to be very sensitively done… From my experience, older people are still very cautious and very wary of people getting involved, worrying about people interfering with their independence and giving too much information about themselves away

(HSC07, Community Liaison Manager)

They would have to use lay language. It's no good asking somebody, ‘Are you depressed?’ Because very few people would say yes. Unless you were very far gone. They might say yes if they were very far gone. ‘It's a depression’, but most of them wouldn't want to admit it.

(OA09, 85 years old)

The approach that the FRS used to discuss mental health during observed HFSVs varied and reiterated some of the concerns raised by participants, as recorded in the following observations.

Technician then moved onto asking more about wellness/well‐being.

– ‘How is your mental health?’ [OA2: fine, yes]. OA2 didn't really say much to that. OA2 was not phased by the question;
– ‘do you ever feel depressed?’ [OA2: oh no. no.];
– ‘do you ever feel lonely?’ [OA2: yes, a lot of the time, especially recently with this pandemic. I'm sick of myself a lot of the time. Sick of my own company]. Technician asked if she had visitors or if there was any family around and OA2 explained her son and daughter live relatively nearby and her daughter popped in about twice a week. Technician asked if her OA2 could ask her daughter to check the alarms more frequently, at least once a month. OA2 agreed.

CSO approached the question stating that the last couple of years have been very tough for a lot of people. Mental health is something that we don't talk about very often but is very important. He also referenced OA13's recent health challenges and asked how they were both doing, were they coping and how was their mental well‐being.

(Observation data 18 October 2022)

The inconsistencies (direct, closed questions vs. conversational open questions) in the approach to discussing mental health with service users illustrates the need for additional training to aid consistent approaches and to support FRS staff confidence to respond appropriately to the disclosure of struggles by service users.

### 3.2 Safely Identifying Mental Health Problems

#### 3.2.1 Opening a ‘Can of Worms’

While HSC participants broadly accepted and supported the proposed expansion of FRS HFSV to include more focus on anxiety and depression in older adults. However, some were concerned that, without appropriate and sufficient training, FRS staff could ‘open a can of worms’ (HSC04, Community Matron; HSC06 Social Worker).

I think the person's got to have some training and also support for them as well because they could open a can of worms and go into somebody who's suicidal and they've got to be able to know how to respond haven't they?

(HSC04, Community Matron)

FRS would need to carefully find the balance between asking the right questions in an appropriate style, while not damaging trust and rapport they may already have established with the service user. It has been suggested that questions should be posed in a conversational style:

I think they would but you need to do it in the right way and that's what I was saying about the training being very important because if you've got a GP that goes in and starts asking those questions, I think an older person will expect it… I think you can have a tick box because it's relevant as you need to collate information but I think you don't have to give them a tick box or go through a questionnaire. That questionnaire should be a conversation and that tool should only be for the person that's asking the questions.

(HSC16, Psychotherapist)

Older adults varied in their opinion of the FRS asking about mental health symptoms. One older adult, a retired mental health nurse, suggested mental health experts should be involved in the delivering training to FRS to support difficult conversations with older adults:

I think it would need the training. It depends who does the training. I think you need experts in mental health. I can't see a problem in them discussing physical problems, like mobility, because people will look for help and they're glad to talk about it. Mental health is just that thing that nobody wants to talk about.

(OA01, 67 years old)

#### 3.2.2 Preparing the Public in Advance

Many (HSC and older adults) felt that it would be appropriate for the FRS to utilise their time during the HFSVs to cover anxiety and depression. However, participants recognised a need to prepare members of the public, especially older adults, in advance about the inclusion of potentially stigmatising topics of conversation (i.e. mental health). A letter ahead of a HSFV and/or media content was suggested.

I suppose part of it is about the branding of the visit, but also maybe being upfront about the types of questions that are going to be asked

(HSC08, GP)

Social media was deemed acceptable by one older adult, although they acknowledged that some people do not use social media:

I mean, Facebook is quite a good one, if you can get on to a local … but then again, how many elderly people do Facebook?

(OA11, 67 years old)

However, most older adults interviewed felt that leaflets were often forgotten about or discarded.

Me, for example, my recycling bin for paper is right by my front door. [Yeah]. So junk mail comes through, you know… I mean, yeah, leaflets, as you say, you know, would people look at them?

(OA11, 67 years old)

Technician handed the paperwork (form from the visit, and the user manuals for the new alarms) to OA8. OA8 placed them in the side of his wheelchair and the paperwork proceeded to slip through a gap between the cushion and the side of the chair. He laughed and said ‘do you like my filing system?!’. We all laughed at this and technician offered to place the paperwork in a neat pile on a sideboard in the corner of the living room.

(Observation data 15 July 2022)

Older adults suggested that avoiding using the term ‘mental health’, ‘anxiety’ and ‘depression’ might help the FRS maintain rapport with older adults who may not find this language acceptable:

They would have to use lay language. It's no good asking somebody, ‘Are you depressed?’ because very few people would say yes. Unless you were very far gone. They might say yes if they were very far gone. ‘It's a depression’, but most of them wouldn't want to admit it.

(OA09, 85 years old)

However, one older adult suggested that avoiding using language such as ‘feeling sad’ and ‘low mood’ may not be inclusive language, especially for someone who is living with depression and/or open to using the medical terminology of ‘anxiety’ and ‘depression’:

I think, again, low mood could just be somebody who's having an off day. Or you could look at the weather and think, ‘Oh, it's really depressing, this weather, isn't it?’. So that's low mood. But for somebody who's really struggling, low mood could be a good day for them, couldn't it?

(OA08, 67 years old)

#### 3.2.3 In Need of Training

Training needs were identified by participants to deliver extended HSFVs; including mental health awareness, communication skills and aspects of self‐care for FRS staff to avoid being impacted by difficult and distressing experiences. Many HSC providers described that FRS needed mental health awareness training:

*The difficulty is not about asking the question, the difficulty is knowing what to do with an answer… I think they might struggle because if you are not trained these kinds of sensitive things could be quite distressing for the fire team as well to be with mental health* (HSC09, GP). HSC providers suggested that the FRS would need to have knowledge of resources in their local area that could support mental health among older adults:

It's about having those tools. I think one of the really important things is to have the right signposting for each area that they're obviously going into and varying what they do and which would be the right service to advise them to maybe get in touch with.

(HSC16, Psychotherapist)

One older adult suggested that FRS staff should be given training to support delivery more intensive support, as opposed to just sign‐posting, to avoid a sense of uncertainty for both provider and recipient and address existing service access challenges.

If the fire person was given the framework and resources to do the next stage, to make it work, otherwise you have the older person who thinks ‘well no body came’, and you've also got the firefighter thinking ‘Oh, well, I hope I did that right, but I know that there aren't enough telephone support workers from whatever’.

(OA07, 73)

The observations identified areas in which training on mental health awareness and communication may be of benefit to the way in which the topic is approached to better support older people disclosing struggles.

A FRS Technician asked the questions on his form about mental health. The conversation went as follows:

– ‘How's your mental health?’ [OA07: yea, fine]
– ‘Any depression?’ [OA07: no]
– ‘Loneliness?’ [OA07: I never used to be but since my wife died, I do miss having someone to talk to and I do get lonely].

Technician responded to say that he was sorry for his loss and moved onto the next question.

(Observation data 13 July 2022)

#### 3.2.4 Making Space for More Referrals

Some HSC participants expressed concerns about service‐level capacity; they were wary of added further pressures onto already overwhelmed primary and social care systems:

I would say no [laughter]. To me, it seems like there's no capacity in mental health services because I rarely get a response to a referral but I don't work in mental health services. Everyone is overstretched, aren't they?

(HSC05, Community Matron)

One GP stated that while they thought the intervention was a good idea, they were concerned about the potential increase in referrals to GPs and subsequent workload.

I think 100% we will support it. The only challenge and the dilemma to this is about the time and the funding… The last thing the GPs would want is to be stretched and be given extra additional work with no time or dedicated funding for it.

(HSC09, General Practitioner)

Meanwhile, another providers commented that an increase in referrals may highlight the need for additional funding from local commissioning bodies:

I think it would identify more and hopefully by identifying this pocket of people it might help to improve the services that are out there. You know, we might be able to get some more funding.

(HSC04, Community Matron)

A Community Nurse stated that while they already have very high workloads, they would always make room for those who need the care and support where it is needed.

Well, I think it would identify more and hopefully by identifying this pocket of people it might help to improve the services that are out there. You know, we might be able to get some more funding.

(HSC04, Community Matron)

## 4 Discussion

### 4.1 Summary of Findings

There was broad‐level support from interview participants for the FRS to expand its HFSVs to include greater emphasis on mental health. Having ‘a foot in the door’ with people in underserved groups, out in the community, offers an important opportunity that other services lack. The FRS can act as ‘An extra pair of eyes’, particularly for services that do not offer home visits, to witness home environments and risks. Home visits were seen to provide older adults a ‘Time to talk’ with a trusted professional. HSFVs usually last between 30 min to an hour but can take longer depending on level of need. The FRS, therefore, have more time to talk to service users than some other HSC partners.

Study participants broadly supported FRS staff to adopt a light‐touch approach to identify signs of anxiety and depression and to receive adequate training to manage conversations to prevent ‘Open[ing] a can of worms’. All providers felt training for FRS was necessary to support communication skills, partnership working and self‐care. HSC participants expressed concerns about capacity in primary and social care to cope with extra referrals; though a mixed view was taken towards this with an increase in referrals perceived as an opportunity to support prioritisation and funding. Observations demonstrated that it would be feasible for the FRS to include more on mental health throughout their HFSVs and training would be necessary to deliver this.

Findings suggested that for the community, public awareness campaigns would be needed to highlight the changes to the HSFVs; this would help to prepare older adults to have conversations about non‐fire‐related topics. The language of mental health was considered by participants with some older adults preferring terms, such as, coping, worry and sadness rather than medical diagnoses (anxiety, depression) to lessen stigma. However, other older adults felt that avoiding using ‘depression’ and ‘anxiety’ may perpetuate the stigma around mental health. This was reiterated by some of the PAG contributors. Although, this raises questions about whether mental health services have sufficient capacity to identify needs in meaningful timescales.

#### 4.1.1 Comparison With Existing Literature

Previous research identified the FRS as a suitable non‐traditional provider of health care [18]; the FRS have historically supported other services, such as, the NHS Ambulance Service to help deliver care and relieve pressure on system‐level capacity [21, 29, 30]. Our findings support the view that FRSs could play a vital role in identifying signs of anxiety and depression in older adults and provide low‐level support through signposting.

Older adults are commonly perceived by healthcare professionals as reluctant to discuss mental health and that this contributes to the challenges of identification of anxiety and depression [11]. Our data indicate that older adults would be open to discussing mental health a person that they trust, such as FRS staff, particularly where conversations are approached using more familiar and less stigmatising language such as coping, worry and mood. This reflects findings reported elsewhere [31]. The current study identified specific training needs for FRS, as described by HSC and older adult participants. Training needs were also raised by FRS staff in a parallel study [23]. It is apparent that conversations about mental health need to be treated sensitively due, primarily, to the potential for stigma but also to avoid damaging the trust and rapport established in the relationship between FRS and the public. Trust is critical to the acceptance of advice and referrals that FRS staff would make, as highlighted in similar work in mental health involving social prescribing and link workers [32].

The development of partnership working is a key target for the Integrated Care Boards [33]. The current study, in conjunction with Fisher et al. [23], demonstrates the desirability within the FRS, HSC providers and recipients of care for the FRS to work collaboratively with HSC partners to support the identification of older adults showing signs of anxiety and depression and directing them to relevant support agencies. However, attention should be given to ensuring that existing mental health services have the capacity and commitment to act on FRS referrals to ensure that any referral raised is dealt with by trained mental health professionals, rather than emotional burden for the FRS staff who remain the last eyes to see the service user.

The HFSVs would seem to represent an important opportunity for behaviour change in context of mental health; this complements the ‘Making Every Contact Count’ (MECC) approach and its key principles: empowerment, person‐centred and brief conversations [34]. A recent systematic review of MECC‐informed interventions highlighted poor theoretical underpinning in behaviour change [35]. Thus, any future interventions delivered by FRS should be designed with theory in mind if it is to maximise this opportunity for contact.

### 4.2 Strengths and Limitations

This paper reports the views of HSC providers and older adults about extending the role of FRS to incorporate mental health checks and guidance. HSC participants were diverse in occupation, length of service, seniority and age. Older adults in both interviews and observations were diverse in age, geographical location and living situation, but there was no ethnic diversity among the participants. The poor ethnic diversity reflects the demographic profile of the area and the services involved. This study took place in a single region of the United Kingdom; findings may not be transferable to other services (HFSVs are delivered by most FRS, however the delivery differs by locality depending on governance structures) and further research and flexibility is needed to understand how FRS‐led mental health interventions can be optimised through design and implementation.

We excluded four participants as these were considered ‘imposter participants’ [28]. Each of these were recruited via social media. We and colleagues have since used social media with caution when recruiting participants. We also developed a strategy to monitor all participants recruited online (e.g., asking for date of birth as well as age).

### 4.3 Implications for Practice, Policy and Research

There is scope to develop a training programme for FRS and mental health staff. This would involve local and national FRS co‐producing an intervention to expand the existing HFSV and an evaluation of the implementation of the training and intervention and ensure that it is suitable for national FRSs. In March 2023, the authors hosted a workshop with local and national FRS staff, PPIE contributors, academics, HSC providers and wider members of the public to identify key mental health messages for older people, a set of learning objectives for the proposed FRS training and views on referral pathway design. Discussion at this workshop has informed a further grant development and a YouTube video.

## 5 Conclusions

HSC providers and the FRS [24] agree that the HFSV could be expanded to include questions and conversations that would help identify anxiety and depression in older adults. Expanding the HFSV could support HSC services and primary care to identify anxiety and depression, which could, in turn relieve pressure on wider HSC systems. By providing a face‐to‐face service that many services have reduced, the FRS are able to offer an additional pair of eyes to identify visual signs of mental ill‐health in an underserved population. The FRS could also offer a light‐touch approach to signposting to relevant support agencies. HSC providers mostly agreed that while they may experience some increase in referrals from the FRS, it was not felt that this would overburden an already stretched system and leave individual FRS staff with the concerns of those they feel are vulnerable and require additional support.

## Author Contributions

**Tamsin Fisher:** investigation, writing–original draft, formal analysis, writing–review and editing, project administration, and data curation. **Carolyn A. Chew‐Graham:** conceptualisation, methodology, supervision, formal analysis, project administration, writing–review and editing, funding acquisition, investigation, validation, visualisation, and data curation. **Nadia Corp:** conceptualisation, data curation, formal analysis, writing–review and editing, and funding acquisition. **Saeed Farooq:** conceptualisation, data curation, formal analysis, writing–review and editing, and funding acquisition. **Paul Kingston:** conceptualisation, formal analysis, writing–review and editing, funding acquisition, and data curation. **Ian Read:** writing–review and editing and data curation. **Gary Spolander:** conceptualisation, formal analysis, writing–review and editing, and funding acquisition. **Jane Southam:** writing–review and editing, formal analysis. **Dean Stevens:** writing–review and editing, formal analysis, data curation, and methodology. **Carmel Warren:** writing–review and editing, formal analysis, project administration, and data curation. **Tom Kingstone:** conceptualisation, funding acquisition, writing–original draft, writing–review and editing, methodology, visualisation, formal analysis, project administration, data curation, and supervision.

## Ethics Statement

This study received ethical approval from the Keele University Research Ethics Committee (Ref: MH‐210200).

## Conflicts of Interest

C.A.C.‐G. is an Editor in Chief, HEX. T.K. is a member of the Editorial Board, HEX.

## Supporting information

Supporting information.

## Data Availability

The data that support the findings of this study are available on request from the corresponding author. The data are not publicly available due to privacy or ethical restrictions.
