# Supplementary material for: Defining the Role of the Fire and Rescue Service in Mental Health Support for Older Adults: A Qualitative Study
Source: Health Expect. 2024 Sep 19;27(5):e70028. doi: 10.1111/hex.70028 (PMC11411195; doi:10.1111/hex.70028)
Supplement: Supplementary file 1 — Supporting information. [file HEX-27-e70028-s001.docx]

**FIRESIDE STUDY**

**Home visit observation framework**

**Date of home visit: _________________**

**Consent recorded to observe home visit: 🞎**

| **What to observe?** | **Notes** |
| --- | --- |
| **Neighbourhood location** |  |
| **Home environment** |  |
| **Interaction with Fire and Rescue Service Officer** |  |
| **Level of engagement**  (e.g. receptiveness, asking questions, accepting information) |  |
| **Outcome / plan of action** |  |
| **Timings** |  |
| **Anything else?** |  |
